# Enhancing Urban Wastewater Treatment through Isolated *Chlorella* Strain-Based Phytoremediation in Centrate Stream: An Analysis of Algae Morpho-Physiology and Nutrients Removal Efficiency

**DOI:** 10.3390/plants12051027

**Published:** 2023-02-24

**Authors:** Costanza Baldisserotto, Sara Demaria, Michela Arcidiacono, Elisa Benà, Pierluigi Giacò, Roberta Marchesini, Lorenzo Ferroni, Linda Benetti, Marcello Zanella, Alessio Benini, Simonetta Pancaldi

**Affiliations:** 1 Department of Environmental and Prevention Sciences, University of Ferrara, C.so Ercole I d’Este, 32, 44121 Ferrara, Italy; 2 HERA SpA—Direzione Acqua, Via C. Diana, 40, Cassana, 44044 Ferrara, Italy; 3 Terra&Acqua Tech Laboratory, Technopole of the University of Ferrara, Via Saragat, 13, 44122 Ferrara, Italy

**Keywords:** nutrient removal, nitrogen, phosphorus, urban wastewaters, centrate, native microalgae

## Abstract

The release of inadequately treated urban wastewater is the main cause of environmental pollution of aquatic ecosystems. Among efficient and environmentally friendly technologies to improve the remediation process, those based on microalgae represent an attractive alternative due to the potential of microalgae to remove nitrogen (N) and phosphorus (P) from wastewaters. In this work, microalgae were isolated from the centrate stream of an urban wastewater treatment plant and a native *Chlorella*-like species was selected for studies on nutrient removal from centrate streams. Comparative experiments were set up using 100% centrate and BG11 synthetic medium, modified with the same N and P as the effluent. Since microalgal growth in 100% effluent was inhibited, cultivation of microalgae was performed by mixing tap-freshwater with centrate at increasing percentages (50%, 60%, 70%, and 80%). While algal biomass and nutrient removal was little affected by the differently diluted effluent, morpho-physiological parameters (*F_V_*/*F_M_* ratio, carotenoids, chloroplast ultrastructure) showed that cell stress increased with increasing amounts of centrate. However, the production of an algal biomass enriched in carotenoids and P, together with N and P abatement in the effluent, supports promising microalgae applications that combine centrate remediation with the production of compounds of biotechnological interest; for example, for organic agriculture.

## 1. Introduction

Water pollution is related to the growth of population and industrialization [1]. About 30% of the world’s freshwater resources are used by industry and urban settlements, which in turn produce high amounts of wastewaters containing several chemicals at various concentrations [2]. The composition of urban wastewaters (UWW) depends on the behavior, standard of living, and lifestyle of inhabitants, and on laws governing the local activities [3]. UWWs are characterized by biodegradable organic matter, nutrients, heavy metals, hormones, detergents, pharmaceuticals, pesticides, fats, oils, pathogenic microorganisms, and several other contaminants [3,4]. The high concentrations of nutrients in inorganic form, i.e., nitrogen (N) as ammonium and phosphorus (P) as orthophosphates, are due to the degradation of urea and proteins and to the microbial degradation of organic phosphates and polyphosphates, respectively [5,6]. The release of wastewater into waterbodies causes various imbalances in aquatic ecosystems, e.g., eutrophication, which can produce loss in water quality and risks to human health [7]. To avoid eutrophication, Europe Directives 91/271/EC and 98/15/EEC establish the allowed nutrients level before treated wastewater can be discharged (European Union Legislation) [8,9]. In this regard, legislation is available worldwide to limit the same problems [10,11]: as an example, in the USA, the Environmental Protection Agency developed the NPDES permitting system as a response to the serious environmental degradation that was occurring before its introduction [12]. Wastewater treatment plants (WWTPs) are used to remove nutrients and organic matter through physical, chemical, and biological processes [13]. Commonly, biological treatments, in which ammonium is converted to gaseous nitrogen by a combination of aerobic and anaerobic processes, are widely used for nitrogen removal. However, these processes involve the production of large quantities of waste sludge, as well as high costs and complex operativity as side effects [13,14]. Similarly, the process typically used to remove phosphates in many WWTPs is the chemical precipitation of P, but this process requires the addition of high concentrations of metal coagulants which can alter the biological activity of activated sludge and contribute to increase pollutants in the sludge [15]. Although depurated water outflow from WWTPs is not an environmental problem, some wastewater streams within the process still contain high concentrations of N and P, mainly in the form of ammonium and phosphates. Therefore, these streams are usually recycled in the WWTPs for further treatments, thus increasing the management costs of the treatment plants [16]. The stream from the dewatering process of anaerobically digested sludges (the so-called “centrate”) is one of the cases where further treatment is required due to the presence of high nutrient concentrations [16,17]. Nevertheless, owing to its high content of carbon, nutrients, and various minerals, centrate is a potentially suitable substrate for microalgae cultivation [16,18,19]. In fact, microalgae, i.e., unicellular photosynthetic organisms well-known for their rapid growth, high biomass productivity and remarkable ability of CO_2_ sequestration, can grow in substrates rich in both organic and inorganic compounds, such as urban wastewaters [20,21,22,23,24]. Since microalgae assimilate and consume N- and P-containing inorganic compounds to grow, the introduction of a microalgae-based system as tertiary treatment in a WWTP is considered an economical and environmentally friendly alternative way for nutrients removal from wastewaters [25]. In addition, microalgae can also remove micropollutants, such as heavy metals, and persistent organic contaminants, such as chlorinated hydrocarbons, dyes from textile industries, and herbicides, which are not sufficiently removed by conventional treatment processes [26,27,28,29,30]. Interestingly, in addition to the phytoremediation effect, wastewater treatment using microalgae would lead to the production of algal biomass that can be used as a source of value-added products in the energy, nutraceutical, agricultural and feed sectors [31,32], thus improving the economics of both microalgal biomass obtainment and of remediation costs [33]. However, not all algal species are equally capable to remove N and P, and to adapt their growth in wastewaters. Therefore, selection of microalgal strains is critical to develop effective nutrients abatement systems [26]. Since several species of microalgae are naturally present in wastewaters, their isolation could be a successful approach to obtain microalgal strains suitable for the treatment of such waste matrices [13,18,26,34,35,36]. In fact, it was demonstrated that native microalgae can achieve higher nutrient removal rates than commercial ones, since they are already adapted to wastewater conditions, and are more resilient to fluctuations of wastewater composition and to the presence of other microorganisms [13,36,37,38].

The operational fluctuations in WWTPs, together with the variability of centrate composition and of its physico-chemical characteristics due, for example, to the lifestyle of the inhabitants, represent a challenge for microalgae cultivation in this substrate. Moreover, the presence of heavy metals and other compounds, such as polyelectrolytes (used to improve solid–liquid separation during the dewatering process) and organic acids, along with high nutrient concentrations in this kind of effluent may have negative effects on the success of microalgae cultivation [7,39]. Therefore, investigating the optimal concentrations of centrate is of great importance in view of its employment in an efficient system which combines microalgae-driven bioremediation and economically viable production of algal biomass [19,40,41,42].

The aim of this work was to isolate a native and promising microalgal strain from the centrate stream of the digested sludge dewatering process of the local WWTP for its potential subsequent use in a prototypical phytodepuration plant to be integrated in the same WWTP or in other plants located in the geographical area of Ferrara, thus limiting problems linked to possible floristic contaminations [43]. In this regard, growth and photosynthetic responses of the isolated microalgal strain chosen for phytoremediation tests were first compared using centrate streams, as cultivation media, or a synthetic medium with the same N and P concentrations as those present in the effluent. Then, growth, morphology, photosynthetic pigments content, photosystem II (PSII) maximum quantum yield and nutrients removal ability of the microalgal isolate were tested in centrate effluent diluted with freshwater at different percentages, from 50 to 80%. Since the present research was designed as a preliminary necessary step to plan a possible scale-up of the process, the present experimentation was conducted on a laboratory scale.

## 2. Results

### 2.1. Isolation and Selection of an Autochthonous Microalgal Strain from Centrate Stream

**Figure 1:**
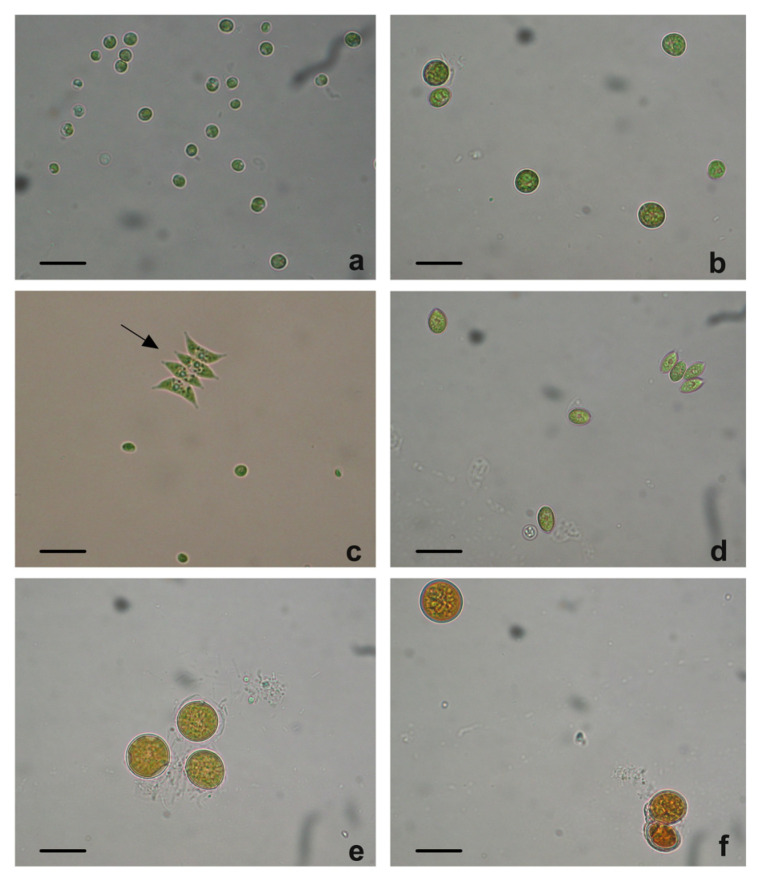
Light microscope images of the most representative microalgal strains collected from the urban wastewater (UWW) stream deriving from the dewatering process of digested sludges (=centrate). Images refer to: (**a**) roundish green cells identified as *Chlorella* sp.; (**b**) isolated strain characterized by larger roundish green cells (cell diameter of about 10–15 µm); (**c**) mixed culture showing small green roundish cells and a typical colony of *Scenedesmus* sp. algae (arrow); (**d**) strain of microalgae identified as *Scenedesmus* sp.; (**e**,**f**) microalgal isolates characterized by large (20 µm) greenish to orange cells. Bars: 25 µm.

In the first step of this work, the isolation and selection of autochthonous microalgal strains from centrate samples were performed. As shown in Figure 1, several species of microalgae were found in the effluent.

Among the isolated microalgal strains, the most common forms were roundish green cells (Figure 1a,b), characterized by a cell diameter ranging from about 3–5 µm (Figure 1a) to 10–15 µm (Figure 1b). Besides these microalgae, cells with an elongated shape (major axis: 10–20 µm) (Figure 1c,d), and large roundish cells (diameter: around 20 µm) (Figure 1e,f) were also found. Based on morphological aspects, it was possible to identify some of the strains isolated as belonging to genera *Chlorella* (Figure 1a) and *Scenedesmus* (Figure 1c,d; in c, consider the algae indicated by the arrow). As in the present work the isolation of native strains was aimed at obtaining microalgae cultures that were dense enough to be used to phytoremediate centrate wastewater effluents, all strains were preliminarily cultivated in a synthetic medium used to boost algal growth and, thus, to allow the production of sufficient algal biomass for further tests. Algae ascribable to *Chorella* sp. cells, i.e., those shown in Figure 1a, grew better than the other isolates (not shown); therefore, that strain was chosen for further trials of cultivation in the effluent and of nutrient removal. Identification of the selected strain as *Chorella* sp. cells was further supported by the transmission electron microscopy observation of the characteristic chloroplast containing a large pyrenoid and stromatic starch granules (Figure S1).

### 2.2. Preliminary Growth Tests of Chlorella Isolate in Centrate Stream and Modified BG11 Medium

#### Growth Kinetics and PSII Maximum Quantum Yield

**Figure 2:**
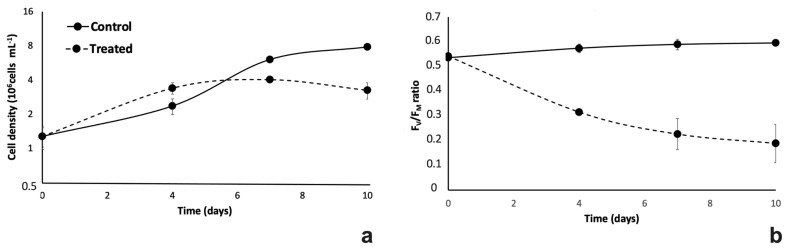
Growth kinetics (**a**) and photosystem II (PSII) maximum quantum yield (*F_V_*/*F_M_* ratio) (**b**) of *Chlorella* sp. cells cultivated in modified synthetic BG11 medium (control samples; black line) and in 100% centrate (treated samples; dotted line). For growth kinetics, cell densities (10^6^ cells mL^−1^) are plotted on a base 2 logarithmic scale. For each sample, values are means ± standard deviations (s.d.; *n* = 3 for cell density; *n* = 6 for *F_V_*/*F_M_* ratio). In both cases, except for inoculation time (time 0), differences between control and treated samples were always significant (Student’s *t* test; *p* < 0.05).

To assess growth ability in the effluent, the selected *Chlorella* sp. was cultivated in 100% centrate and in a synthetic modified BG11 medium containing the same concentrations of N and P as those present in the effluent; cultures in synthetic medium were kept as controls. In Figure 2, growth kinetics and PSII maximum quantum yields of microalgal strains cultivated in both conditions are reported. Although both cultivations started with the same cell density (ca. 1.3 × 10^6^ cell mL^−1^), growth curves showed an evident different trend (Figure 2a). A typical growth curve consisting of lag, exponential, and stationary phases was observed in the controls. In detail, after a 4-day-long adaptation period, the exponential growth phase lasted until day 7, when the cell density reached 6 × 10^6^ cell mL^−1^. In this phase, the cells were characterized by a growth rate of 0.45 d^−1^. After the exponential growth phase, control cultures entered the stationary phase, reaching 8 × 10^6^ cell mL^−1^ after 10 days of cultivation. In contrast, no lag phase was observed in the centrate-treated cultures, which instead showed a prompt exponential phase that lasted until day 4 of growth, when the cell density reached values of about 4 × 10^6^ cell mL^−1^, 43% higher than that in control samples (Figure 2a). Nevertheless, in the exponential growth phase, cells in centrate effluent were characterized by a growth rate lower than that observed in control cultures (ca. 0.2 *vs.* 0.45 d^−1^ for treated and control samples, respectively; *p* = 0.017), and after day 4, the kinetics showed a decline phase, characterized by a progressive, slight decrease in cell density down to 3.2 × 10^6^ cells mL^−1^.

As regards the photosynthetic efficiency evaluated as *F_V_*/*F_M_* ratio, i.e., the maximum quantum yield of photosystem II (PSII), starting from a value of about 0.57 in both cultures at time 0, in controls the *F_V_*/*F_M_* ratio reached values next to 0.6 during the first 4 days of cultivation, and then remained quite stable up to the end of the experiment (10 days) (Figure 2b). Differently, in treated samples, the PSII maximum quantum yield did not maintain values around 0.6 during the cultivation period, but had evidently dropped to values around 0.3 by day 4, and then continued to decrease to 0.2 by the end of the experiment (Figure 2b).

### 2.3. Phytoremediation Experiment in Diluted Centrate Stream

#### 2.3.1. Algal Growth Aspects

**Figure 3:**
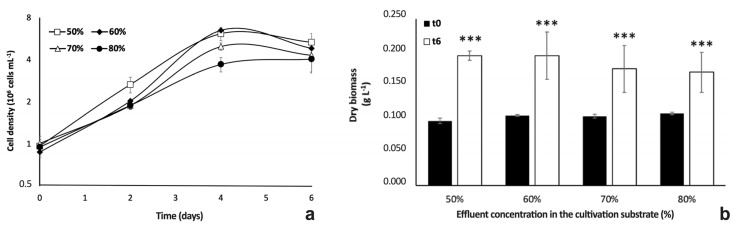
Growth kinetics (**a**) and dry biomass yields (**b**) of *Chlorella* sp. cells cultivated in centrate stream at 4 different concentrations: 80%, 70%, 60%, and 50% (percentage of effluent in freshwater). For growth kinetics, cell densities (10^6^ cells mL^−1^) are plotted on a base 2 logarithmic scale. In (**a**) timecourse variations of cell density of algae cultivated in diluted centrate at 50% (empty squares), 60% (black filled diamonds), 70% (empty triangles), and 80% (black filled circles). In (**b**) dry biomass yield (gL^−1^) of cultures at the inoculation time (black histograms), and 6 days after inoculation time (white histograms). For each sample, values are means ± s.d. (*n* = 3). In the case of cell density (**a**), the differences among cultures are significant for day 2 and day 4 after inoculation (one-way ANOVA at: t2, F(3,8) = 23.176, *p* < 0.001; t4, F(3,8) = 49.715, *p* < 0.001; t6, F (3,8) = 3.014, *p* = 0.0941). In the case of dry biomass yield (**b**), differences between inoculation time and day 6 are significant for all cultures (two-tails Student’s *t* test; *** *p* < 0.001).

Results obtained from preliminary cultivation tests suggested that the microalgal growth was somehow inhibited by 100% effluent (Figure 2a). Therefore, the stream was diluted in order to obtain a substrate possibly more suitable for the microalgal growth. Figure 3 shows the growth kinetics of *Chlorella* cells in centrate effluent, diluted with tap freshwater at four increasing concentrations (50%, 60%, 70%, and 80% of effluent).

Although at the end of the experiment (day 6) all cultures were characterized by similar cell densities, algae samples showed a different growth kinetics in response to the effluent concentrations (Figure 3a). Starting from cultures with the same density of algae at time 0 (ca. 1 × 10^6^ cells mL^−1^), all samples immediately entered the exponential phase of growth, which lasted up to day 4 of cultivation, when cultures were characterized by significantly different cell concentrations (6.15, 6.49, 4.98 and 3.71 × 10^6^cells mL^−1^ for cultures in 50, 60, 70, and 80% effluent, respectively; one-way ANOVA, *p* < 0.001—F(3,8) = 49.715). In detail, the highest cell densities were found in cultures with the most diluted substrates (50% and 60%). These results were linked to growth rates ranging around 0.67–0.72 d^−1^ in 50 and 60% wastewater samples, while gradually lower values were recorded in algae samples cultivated in 70% and 80% effluent (0.58 and 0.47 d^−1^ for 70 and 80% samples, respectively; one-way ANOVA, *p* < 0.001—F(3,8) = 18.160). At the 6th day of cultivation (end of experiment), cultures in 80% wastewater reached an evident stationary phase of growth, while cell density of cultures in the other wastewater-diluted substrates tended to decrease, reaching values next to those of the samples in 80% centrate (5.34, 4.83, 4.31, and 4.03 × 10^6^ cells mL^−1^ for cultures in 50, 60, 70 and 80% effluent, respectively; one-way ANOVA; *p* > 0.05—F (3,8) = 3.014, *p* = 0.0941) (Figure 3a).

As regards dry biomass yield, starting from 0.1 g L^−1^ at the inoculation day (time 0), after 6 days of cultivation the cultures with 50% and 60% centrate reached the same values (about 0.2 g L^−1^), while those with 70% and 80% centrate had slightly lower values, respectively, of 0.18 and 0.17 g L^−1^ (Figure 3b). Overall, the final values of dry biomass yield were never significantly different among the four algae samples (one-way ANOVA, *p* > 0.05; F(3,8) = 2.3683, *p* = 0.146).

#### 2.3.2. Morphological Analyses

**Figure 4:**
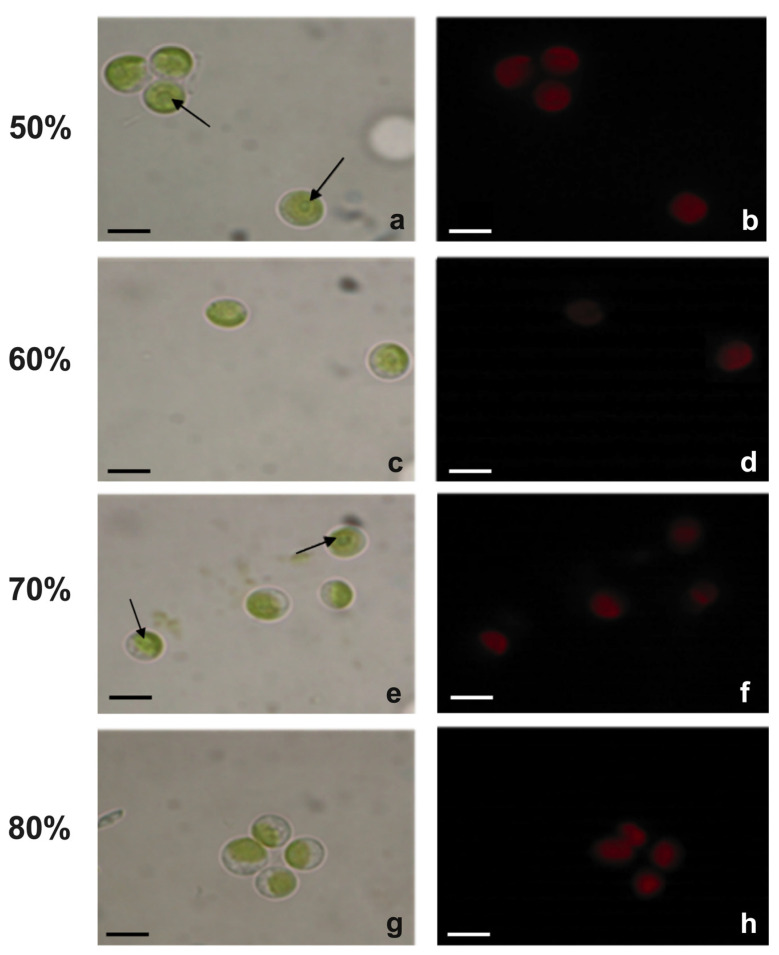
Light and fluorescence microscope images of *Chlorella* sp. cultivated in centrate stream diluted at 4 different concentrations: 50% (**a**,**b**), 60% (**c**,**d**), 70% (**e**,**f**) and 80% (**g**,**h**) of effluent. Images were taken at the end of the experiment (6 days). Figures (**a**,**c**,**e**,**g**) refer to images captured using the white light lamp of the microscope, while Figures (**b**,**d**,**f**,**h**) using the fluorescence lamp (excitation wavelength, 436 nm). Arrow, pyrenoid. Bars, 7 µm.

**Figure 5:**
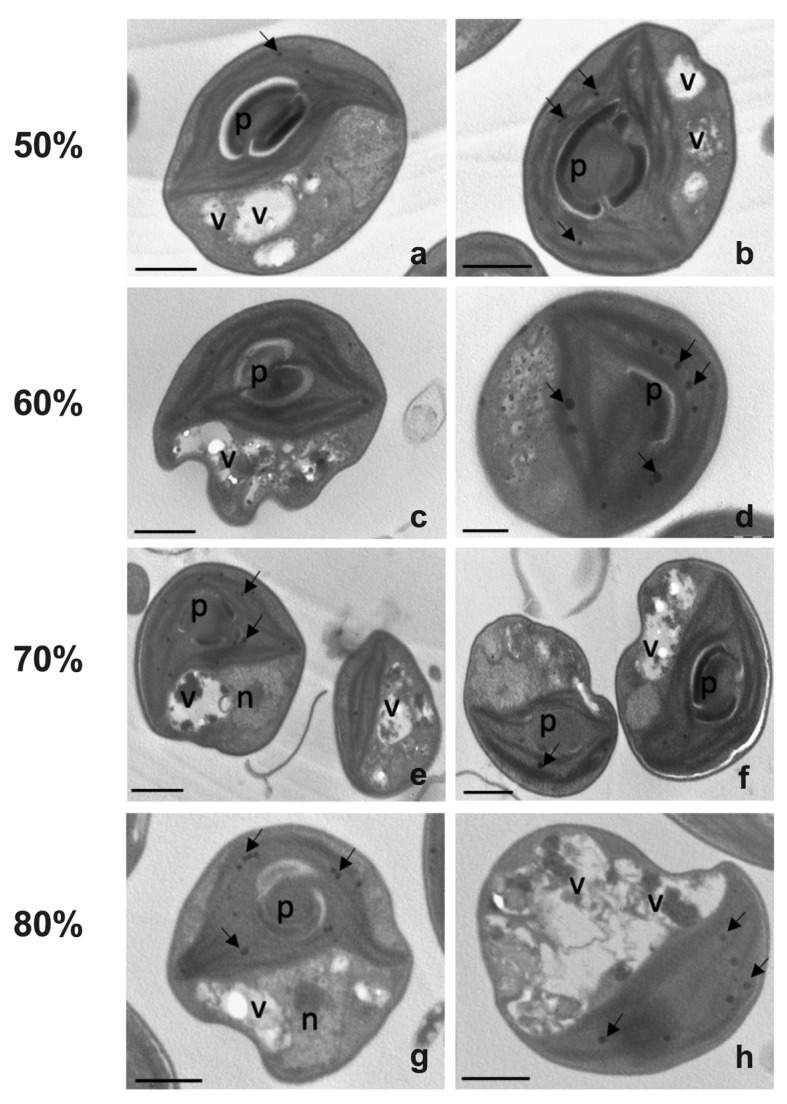
Transmission electron micrographs of *Chlorella* sp. cultivated in centrate stream diluted at 4 different concentrations: 50% (**a**,**b**), 60% (**c**,**d**), 70% (**e**,**f**), and 80% (**g**,**h**) of effluent. Pictures were taken at the end of cultivation (6 days). n, nucleus; p, pyrenoid; v, vacuolization; arrows, plastoglobules. Bars, (**a**–**c**), (**e**–**h**): 1 µm; d: 0.5 µm.

**Figure 6:**
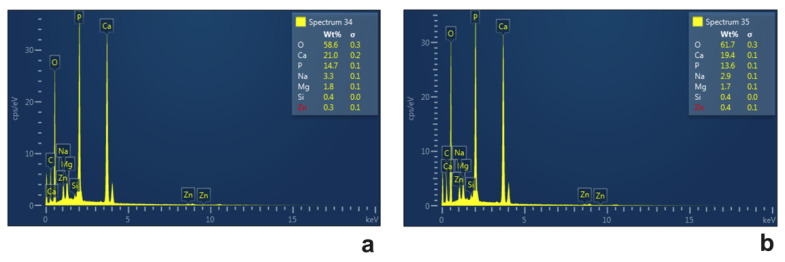
Example spectra of X-ray microanalysis associated with scanning electron microscopy recorded on *Chlorella* sp. cultivated for 6 days in 70%-diluted centrate stream. In (**a**,**b**) two representative spectra recorded from different areas of algae samples are reported. The box at the top right of the spectra images shows the % areas of the elements detected during the analysis.

The cell morphology of *Chlorella* cultivated in diluted effluent was evaluated at the end of the experiment, and images, taken at the light or transmission electron microscope, were compared (Figure 4 and Figure 5). Light microscopy showed that in 50% wastewater cells had a roundish, sometimes slightly flattened, shape with a cell diameter of about 6.2 µm (Figure 4a). A cup-shaped chloroplast, which occupied most of the cell volume and with an evident pyrenoid, was also present inside the cells (Figure 4a). In the other substrates, i.e., those with an effluent percentage from 60% to 80%, the cells showed a progressive decrease in cell size: diameter decreased from 5.6 µm in cells cultivated in 60% centrate to 4.9 µm in those cultivated in 80% centrate (Figure 4c,e,g). In all samples, plastids emitted intense red fluorescence (Figure 4b,d,f,h). The observation of cell ultrastructure provided further details (Figure 5). In all wastewater-cultivated algae, the thylakoids, while appearing appressed, seemed somehow reduced with respect to those in algae cultivated in synthetic medium (Figure S1). As expected, inside the plastid of all algae samples, one big pyrenoid was present; in cells cultivated in 50 to 70% effluent, the pyrenoid was surrounded by abundant starch, typically shaped as a shell around it (Figure 5a–f), and contained crossing thylakoids (Figure 5a–c,e,f). This pyrenoid morphology was less evident in the 80% centrate-grown algae, which also showed more altered plastids overall (Figure 5g,h). Plastoglobules were clearly visible in chloroplasts of all samples (Figure 5). Moreover, vacuolizations were observed in all cells; inside the vacuoles, some dark precipitates were often visible, likely ascribable to polyphosphates depositions (Figure 5). Vacuolization tended to be more evident in the cytoplasm of 80% centrate-cultured cells (Figure 5h). RX-microanalysis showed an evident peak of P in all samples (about 14%), thus supporting the phosphate presence in the cells (Figure 6). In any case, P was the third element detected after the dominant oxygen (about 60%) and calcium (about 20%). In all samples, no lipid globules occurred inside the cells (Figure 4 and Figure 5).

#### 2.3.3. PSII Maximum Quantum Yield and Photosynthetic Pigment Content

**Figure 7:**
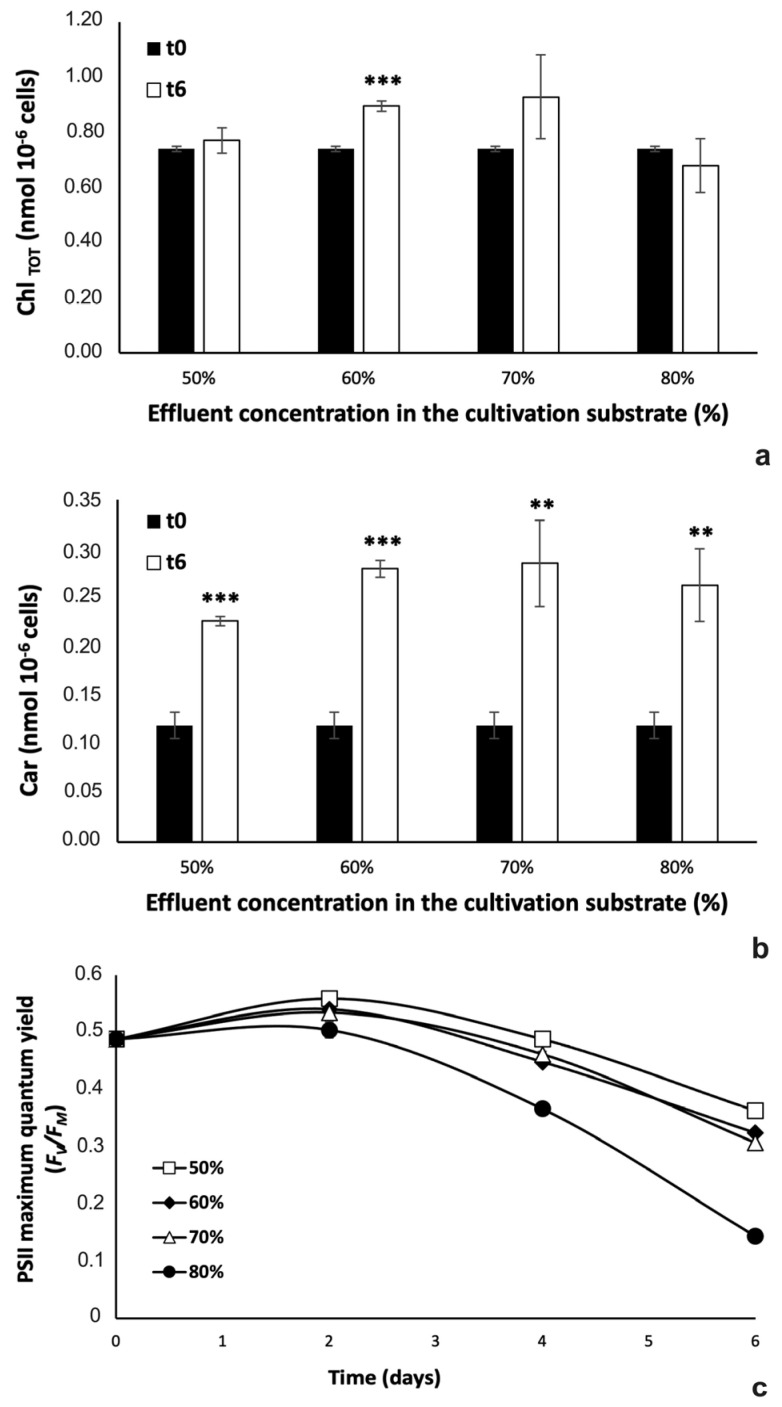
Photosynthetic pigments content (**a**,**b**) and PSII maximum quantum yield (*F_V_*/*F_M_* ratio) (**c**) of *Chlorella* sp. grown in centrate diluted at different concentrations (50, 60, 70, and 80% effluent in freshwater). In (**a**) total chlorophylls (Chl_TOT_) and in (**b**) carotenoids (Car) concentrations are expressed as nmol 10^−6^ cells; black histograms, pigment concentration at the inoculation time (time 0), white histograms pigment content at the 6th day after inoculation. In (**c**), empty squares refer to 50%, black filled diamonds to 60%, empty triangles to 70%, and black filled circles to 80% effluent. For each analysis, values are means ± s.d. (*n* = 3). In (**a**) and in (**b**), two-tails Student’s *t* test compared values at time 0 with those at time 6 days; ** *p* < 0.01; *** *p* < 0.001). In (**c**), at each experimental time, values were compared each other using one-way ANOVA followed by Tukey’s test (*p* < 0.05); for data at 2 and 4 days of cultivation, 60 and 70% samples are not significantly different each other, but are different with respect to both 50 and 80% samples (one-way ANOVA: *p* < 0.001; t2, F(3,8) = 24.95, *p* = 2.05 × 10^−4^; t4, F(3,8) = 502, *p* = 1.91 × 10^−9^), while for time 6 days, differences between all samples are significant (one-way ANOVA, *p* < 0.001; F(3,8) = 1084, *p* = 8.89 × 10^−11^). For simplicity, results of Tukey’s test (=letters for significantly different samples at different experimental times) are not shown.

In order to obtain information about both the physiological state and the biochemical composition of microalgal biomass in response to effluent concentrations, photosynthetic pigment content and PSII maximum quantum yield were evaluated (Figure 7).

As regards photosynthetic pigments, comparing results at the inoculation time with those recorded after 6 days of cultivation in the diluted effluents, the total chlorophylls (Chls_TOT_: Chl *a* + Chl *b*) content significantly increased only in the cells cultured with the substrate containing 60% effluent (two tails Student’s *t* test, *p* < 0.001 for all samples). In the other samples, although the chlorophyll content evidently varied, no significant differences were observed compared to the data from the corresponding time 0 (two tails Student’s *t* test, *p* > 0.05 for all samples, comparing data at t0 and t6) (Figure 7a). Different from what observed for Chls_TOT_, carotenoids strongly accumulated inside microalgae in all cultures, and differences were always significant when comparing data at the inoculation time with those at time 6 days of cultivation (two tails Student’s *t* test, *p* < 0.01 for 50 and 70% samples, and *p* < 0.001 for 50 and 60% ones) (Figure 7b).

Results on PSII maximum quantum yield of algae are reported in Figure 7c. Starting from an *F_V_*/*F_M_* value of 0.48, all cultures showed a slight improvement of the photosynthetic efficiency 2 days after inoculation. Thereafter, a decrease in *F_V_*/*F_M_* values was observed in all samples, especially in cells grown in the 80% effluent; in that case, at the end of the experiment cells reached values lower than those recorded in the other cultures (0.15 in 80% effluent *vs.* 0.36, 0.32 and 0.31 in 50, 60 and 70% effluent, respectively; one-way ANOVA, *p* < 0.001; F(3,8) = 1084, *p* = 8.89 × 10^−11^).

#### 2.3.4. Nutrient Removal from Diluted Centrate Stream

**Figure 8:**
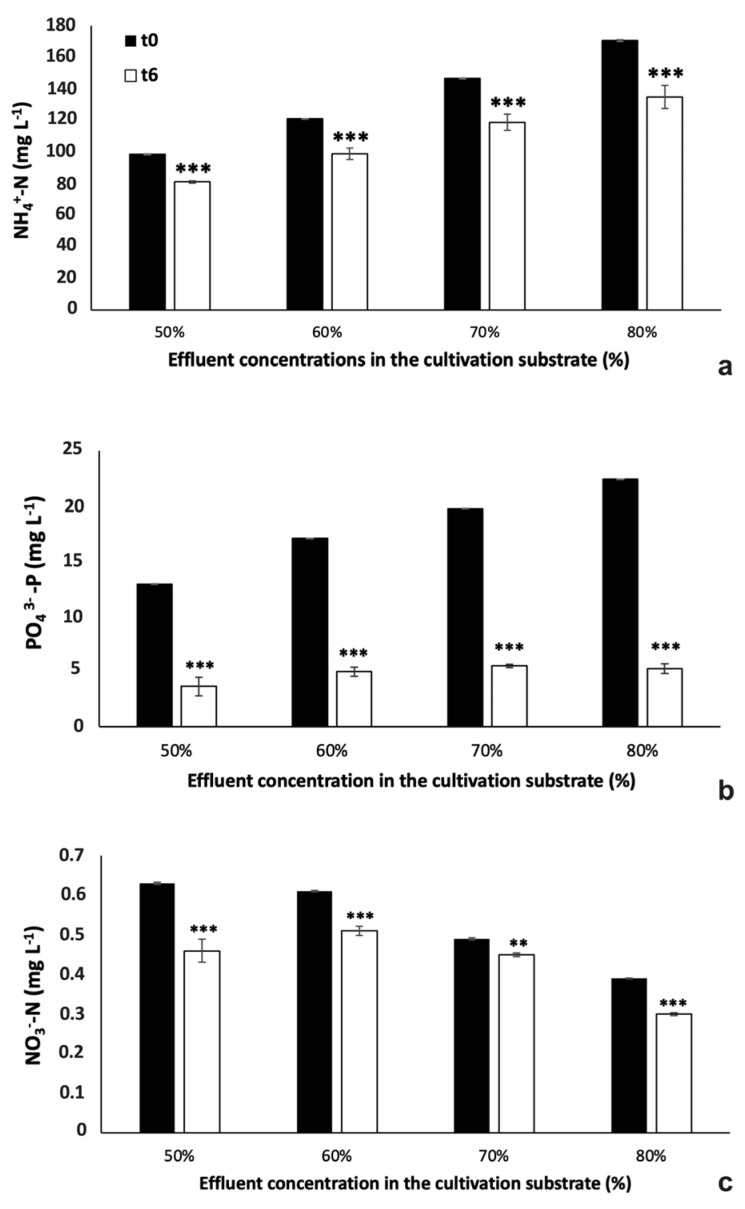
Nutrients concentrations ((**a**) NH_4_^+^-N, (**b**) PO_4_^3−^-P, (**c**) NO_3_^−^-N; mg L^−1^) in winter-season filtered centrate stream, diluted at different concentrations (50%, 60%, 70%, and 80% of effluent in freshwater), at the inoculation time and after 6 days of cultivation of *Chlorella* sp. algae. Black histograms, before inoculation (t = 0); white histograms, 6 days of cultivation (t = 6 days). For each sample, values refer to means ± s.d. (*n* = 3). In all cases, for each sample, two-tails Student’s *t* test was performed comparing concentrations data recorded at time 0 with those at time 6 days. ** *p* < 0.01; *** *p* < 0.001.

For the phytoremediation experiments, parallel to growth and morpho-physiological analysis, nutrients removal from different cultures with diluted effluent was evaluated. Ammonium (NH_4_^+^-N), nitrate (NO_3_^−^-N) and phosphate (PO_4_^3−^-P) concentrations in the effluent before and after microalgae cultivation were compared (Figure 8). During 6 days of cultivation, nutrients concentration was significantly reduced in all samples. As reported in Figure 8a, the NH_4_^+^-N residual concentration was progressively higher as the percentage of wastewater in the cultivation substrate increased, resulting in a similar value of nutrient removal efficiency (RE, %), which was close to 21% for all samples (one-way ANOVA, *p* > 0.05; F(3,8) = 3.01, *p* = 0.094). Differently, PO_4_^3−^-P was around 5 mg L^−1^ in all substrates at the end of experiment, resulting in the most effective abatement of this nutrient in 80% centrate (76.6% RE in 80% effluent *vs.* 72.1, 70.9, and 72.1% RE in 50, 60, and 70% effluents, respectively; ANOVA, *p* < 0.05; F(3,8) = 42.39, *p* = 2.94 × 10^−5^) (Figure 8b).

Despite the filtered centrate had a NO_3_^−^-N content of 0.31 mg L^−1^ (see Paragraph 4.1), the nitrate-N form present in the diluted effluents was the higher, the lower the percentage of effluent in the substrate (0.6, 0.6, 0.5, and 0.4 mg L^−1^ in 50, 60, 70, and 80% effluents, respectively) (Figure 8c). This was a probable consequence of NO_3_^−^-N content in the tap water used for the dilution of centrate (about 0.08 mg L^−1^ in tap water *vs.* 0.03 mg L^−1^ in the centrate used for experiments). Besides this finding, after 6 days of microalgae cultivation, the nitrate concentration in the substrate was reduced depending on its initial concentration, except for the cultures in 50% centrate, where NO_3_^−^-N content was 0.46 ppm, a lower value compared to 0.51 of 60% centrate (Figure 8c). In fact, in this case, the percentages of RE showed that the nitrate abatement was more effective in the 50% centrate with around 27% RE, followed by 23% RE for 80% centrate, 16% RE for 60% centrate and 8% RE for 70% centrate. In any case, these seemingly illogical RE% values may be due to the very low levels of NO_3_^−^-N.

## 3. Discussion

Wastewater treatment plants (WWTP) play an important role in pollutant reduction, public health, and environmental protection by removing biodegradable compounds, nutrients, and pathogens [13,22,44]. Although the use of WWTP is now widespread and proven, over the last years interest has increased in natural methods to efficiently remove excess of nutrients using alternative solutions, including microalgae-based systems [4,33,35,45,46]. Microalgae consume nutrients in the WW to sustain their own growth, at the same time contributing to the overall phytoremediation of the effluent [32,34,35,42]. As commonly occurs in urban WWTPs, also the centrate stream produced in the WWTP of Ferrara (Italy) is also recirculated into the plant because of its high N and P concentration, as well as high BOD_5_ and COD, resulting in additional water-consuming treatments and costs for the management of the whole system. Here, we tested the use of centrate for algae cultivation, in a circular economy view, and found satisfactory conditions for its exploitation.

The microalgae studied in the present work were directly isolated from centrate stream to better cope with potential problems linked to the centrate composition [18,34,37,38]. Of all the obtained isolates, the *Chlorella* sp. one was the most representative, indicating that this isolate was more competitive than the other strains in exploiting the nutrients (and chemical compounds in general) present in the centrate. Previous research reported that algal species belonging to the genus *Chlorella*, usually found in WW treatment ponds, can tolerate and survive in different types of effluent streams [24,34,35,38,47,48,49]. The great competitiveness and extensive use of *Chlorella* sp. in WW remediation, together with the finding that our *Chlorella* isolate showed better growth than the other isolates we had collected, prompted the selection of this strain for subsequent experiments.

Comparative cultivation for 10 d in 100% centrate and in modified BG11 medium showed that the *Chlorella* isolate was able to grow in the centrate stream. Indeed, even if better algae growth and photosynthetic performance were observed in the modified synthetic medium towards the end of cultivation especially, it was noted that growth kinetics of the alga cultivated in 100% centrate (but not in the synthetic medium) was not characterized by a lag phase, thus supporting the idea that the isolate was already adapted to the composition of the effluent and able to exploit it [34]. The rapid initial growth of centrate-treated cultures let them reach a cell density higher than that of controls already at the 4th d of cultivation. Despite this consideration, overall lower growth rate and PSII photochemical yield of algae in 100% centrate stream compared to those of control cultures indicated that the cells were exposed to stress conditions. Indeed, the decrease in *F*_V_/*F*_M_ indicates the damage to the reaction center of PSII, occurring in microalgae exposed to stressful environments [50]. Thus, to limit stressful conditions, we diluted the centrate to find its optimal concentration in phytoremediation tests. Accordingly, although some authors have obtained promising results using 100% centrate for microalgae cultivation [18,34], other researchers reported the need for diluting the effluent because of the inhibitory effect of toxic compounds on algae growth [19,39,40,41,42]. In the present work, results on tests with diluted centrate showed that growth and morpho-physiological aspects of the alga changed in response to increasing concentration of the effluent. As expected, a lower growth rate associated with a progressive decrease in the PSII maximum quantum yield in algae samples grown in 70% and 80% centrate indicated that cell stress increased parallel to the amount of centrate. However, similar biomass yield and cell density were obtained in all cultures at the end of the experiment, suggesting that a late inhibitory effect on microalgal growth occurred in cultures with lower effluent percentages (50 and 60%). Even if various *Chlorella* strains have specific responsiveness to cultivation substrates [17], in this work we found that the overall growth rates of our samples (from 0.7 to 0.47 d^−1^, depending on centrate concentration) in differently diluted centrate were higher than values obtained for other *Chlorella* strains in undiluted centrate as reported by AlMomani and Örmeci in 2016 (0.25 d^−1^) [18], but they were relatively low compared to the those reported by Wang and colleagues (2010) (0.9 d^−1^) in the same type of effluent [34]. Despite differences with the literature data, which were very likely due to both algal strains employed and to different composition of the effluent, it is interesting to note that the present experiment lasted only one week. This specification is noteworthy if one considers that the application of microalgae-based phytoremediation in prototypes or even on real scale is more cost-effective the shorter the cultivation cycles are. In our study, not only the trials had a short duration, but also were linked to promising nutrients removal efficiency percentages (RE%), especially for P.

In the present experimentation, the need to dilute the centrate appeared not to be due to the high concentrations of N and P nor to their molar ratio (in both cases, centrate and modified BG medium shared the same values), but to the presence of other pollutants in the effluent. For example, metal ions are persistent pollutants in nature and also in wastewaters, owing to their non-degradable characteristics, and often make WW unsuitable for microalgal cultivation [51,52]. Although some metals, such as Zn, Mn, Cu, and Fe, are important and essential micronutrients [52], if their concentrations exceed specific thresholds, they become toxic for microalgae [53]. Likewise, their too low concentrations can be limiting for microalgal growth because of their involvement in various metabolic functions [53]. Even if micro- and macro-nutrients such as Mg, Fe, K, Mn, and Ca were not measured in this work, centrate is commonly considered a WW rich in those compounds, essential for algal growth and metabolism [16,17,34]. Thus, it seems reasonable that minerals content in the effluent used for our experiments was suitable for the microalgal growth. On the other hand, other metals, such as heavy metals such as Cr, Ni, Pb, and Hg, are toxic even at low concentrations [54]. In this study, concentrations of these elements in the centrate were in line with those found in the effluent from the thickening step of the same urban WWTP, where heavy metal concentrations did not have an inhibiting effect on the growth of another strain of native *Chlorella* sp. [35]. In addition, a study on *S. obliquus* showed that heavy metals ions inhibited microalgal growth when their concentration was 20 times higher than that found in the effluent used in in this work [55]. However, not only fluorimetric results, but also the analysis of ultrastructure (reduction in thylakoid membranes, large presence of plastoglobules and increased vacuolization as the centrate concentration in the culture medium increased) highlighted a negative effect of centrate on the alga. Alterations of the chloroplast ultrastructure and of the photosynthetic pigments content, together with the decrease in *F_V_*/*F_M_*, are considered as oxidative stress-induced responses [56,57,58]. The oxidative stress in microalgae is promoted by several factors, including chemicals [59]. For example, when present in excess, metal ions alter the activity of antioxidant enzymes and block the electron flow in PSII, leading to intracellular accumulation of reactive oxygen species (ROS) and free radicals [56,60]. Among all metal ions detected in 100% centrate, only Al concentration was similar to those indicated as being responsible for cytotoxic effects (0.4 mg L^−1^) [60,61]. However, the low concentrations of Al reached in the culture medium following dilution of the effluent did not entirely justify the ultrastructural alterations, which were even visible in cells cultivated in 50% centrate. Therefore, it is plausible to assume that such element, in combination with other pollutants, may have increased the stress level in the cells. For example, the presence of acrylamide, a major constituent of flocculants used to improve sludge dewatering efficiency [62], represents for microalgae a stress factor, which results not only in growth inhibition, but also in phenotypical alterations, also in terms of cell size [63]. Furthermore, Costa and colleagues (2014) [64] reported that the uptake of two cationic flocculants and their high affinity for the negatively charged cell surface causes physiological damage in *Pseudokirchneriella subcapitata*.

Another element indicating oxidative stress in *Chlorella* sp. cells was its noticeable increase in carotenoids. In fact, to counteract the damage to biological macromolecules due to stress conditions, microalgae usually increase their content in carotenoids to scavenge ROS and thus preserve the photosynthetic activity [65]. Tolerance mechanisms to WW were already linked to the increase in carotenoids in seven microalgae, including *C. vulgaris*, grown in municipal secondary WW [66]. Furthermore, the stress due to toxic compounds in WW caused remarkable increase in the carotenoids content in *C. sorokiniana* [4]. Although the increase in the carotenoids content is often coupled with lipid accumulation under stress conditions [65], the ultrastructure analysis did not reveal lipid cytoplasmic bodies in any *Chlorella* sample cultivated with centrate, but highlighted the maintenance of starch around the pyrenoid. Intracellular lipid accumulation is a typical response of cells to nutrient starvation [23,67], while starch deposits represent the first carbon storage under mixotrophic conditions [68]. While N and P concentrations in centrate did not indicate a nutrient starvation condition, the COD value highlighted the presence of organic carbon in the effluent, justifying the starch accumulation inside the plastids, as also reported for another *Chlorella* sp. cultivated with urban WW streams [35].

As regards nutrients removal from effluents, it is well-known that microalgal biomass production is accomplished by nutrients uptake from culture medium [26]. In the present study, the low ammonium removal efficiency (about 20%) observed in all cultures was in line with the low biomass production, suggesting that all the ammonium removed from the effluent was assimilated by the cells for biomass production, excluding nitrogen stripping. Furthermore, the decrease in nitrate concentration in 6-day-long experiments also indicated that the nitrification process had not occurred, further confirming that the uptake by the cells to produce biomass was the main phenomenon occurring in the cultures. However, the maintenance of a ca. 20% RE for ammonium in the exhausted substrates with the increase in centrate percentage implicated that available nitrogen exceeded the amount that could be assimilated by the microalgae. Indeed, the ammonium RE% reported in this study was relatively low compared to higher values previously found for other freshwater microalgae in the same type of effluent (N-RE% = from 60 to 100%) [18,19,34,40], or in other urban WW effluents [35]. On the other hand, the phosphate RE% measured for all cultures indicated that P was removed more effectively than nitrogen (about 70–76%, in relation to centrate concentration in the cultivation substrate). This is not so surprising, since it was observed that microalgae can assimilate more P when N concentration is high [69]. Although other Authors reported that an initial N to P molar ratio of ca. 17 as we had in our cultures characterized a P limitation [41,42,70], residual P-PO_4_^3−^ content in the exhausted media in our experiments indicated that the amount of P in the effluent was not limiting for growth. Furthermore, the presence of P as precipitated polyphosphate deposits inside vacuoles (as confirmed by parallel X-ray microanalysis and TEM observations) indicated that P removal was not due to P precipitation in the medium, but that luxury uptake had occurred, so that the algal biomass after phytoremediation was rich in that element.

Overall, our results suggested that the chemical composition of the centrate stream imposed stressful conditions to the microalgae even after dilution. Although the N and P levels at the end of experiments still exceeded the threshold established by the European Directive for water discharge into natural waterbodies [8,9], the use of centrate for *Chlorella* sp. cultivation resulted in a microalgae biomass enriched in both carotenoids and P, whose valorization can contribute to make the algal biomass after WW treatment usable as feed supplement or as organic amendment for soils. Microalgae biomass rich in P, accumulated as polyphosphates thanks to luxury uptake, represents a promising vehicle for returning such element from WW to agroecosystems in the form of biofertilizer [71,72]. As regards carotenoids, the demand for naturally obtained molecules is increasing, thus their extraction from the microalgae biomass after WW remediation processes represents a promising solution [31,32].

## 4. Materials and Methods

### 4.1. Wastewater Collection and Characterization

**Figure 9:**
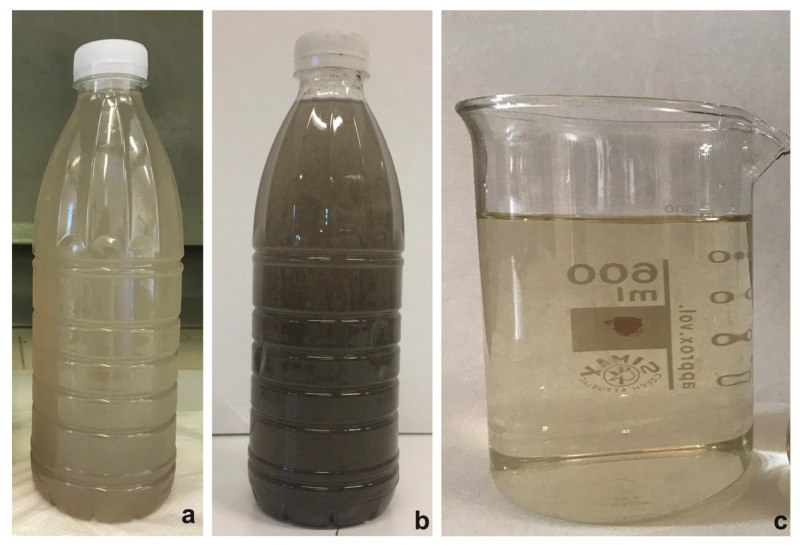
Centrate collected at the HERA-Ferrara wastewater treatment plant after the dewatering process of the digested sludge. (**a**) Centrate stream collected in summertime; (**b**,**c**) Centrate stream collected in wintertime before (**b**) and after (**c**) filtration.

The wastewater employed in the present work was collected after the sludge dewatering process of the WWTP serving the city area of Ferrara, Italy (145,000 population equivalent—PE—on BOD basis; 44°5104900 N, 11°3704700 E), managed by HERA SpA (Holding Energia Risorse Ambiente). In detail, the effluent, called centrate, derived from the centrifugation of anaerobically digested sludge and was sampled in summer and winter season (July 2019 and January 2020). The centrate stream sampled in summer period was quite clear (Figure 9a) and was immediately used to isolate autochthonous microalgal strains (see Section 4.2). Differently, the centrate stream collected during wintertime appeared very dark due to the presence of suspended sludge flakes (OD_750_ was about 0.45) (Figure 9b). Hence, it was first filtered to remove undesirable particles (OD_750_ of filtered samples was 0.005) (Figure 9c), and then it was stored in clean plastic bottles at −20 °C to prevent any physical, chemical, and biological changes before use for microalgal growth and nutrient removal experiments [33]. Filtration removed large-size particles but not all the microorganisms present in the wastewater at the sampling time. Thus, after thawing, the residual bacteria formed a sediment, which was manually removed just before use.

Immediately after harvesting, both centrate streams were analyzed at the internal laboratories of HERA SpA for the content of ammonium (NH_4_^+^-N), nitrate (NO_3_^−^-N), nitrite (NO_2_^−^-N), total phosphorus (TP), COD, BOD_5_, and several metal elements; standard certified methods were used (Table 1). In addition, the NH_4_^+^-N, NO_3_^−^-N, and P in the form of phosphates (PO_4_^3−^ -P) content was also determined in the filtered centrate sampled in winter period (for methods, see Paragraph 4.9; Table 2). It was ascertained that the freezing process did not significantly alter N and P content in the wastewater (data not shown). Traces of acrylamide-based polyelectrolyte flocculant were very likely also present in the effluent due to the use of that compound during sludge centrifugation to improve the separation of solid particles from the aqueous phase.

### 4.2. Isolation and Selection of Autochthonous Microalgae

In order to obtain cultures of single microalgal strains, the centrate stream harvested in July was divided into several jars (150–200 mL volume), containing 100% or freshwater-diluted effluent at the following dilutions: 1:2, 1:3, and 1:5. Subsequently, the jars were exposed to indirect natural sunlight (UVB: 50–200 mW m^−2^; PAR: 150–200 μmol_photons_ m^−2^ s^−1^), at a temperature ranging from 22 to 25 °C, for 6 weeks. After this period, 200 µL of green material grown inside the jars were plated under axenic conditions in Petri dishes containing sterilized BG11 medium (for recipe, please see www.utex.org, accessed on 28 December 2022) with N and P content modified according to the concentrations present in the original wastewaters (Table 1); N source was NH_4_Cl instead of NaNO_3_ used for the preparation of standard BG11. After 4 weeks of incubation, the most dominant single colonies were transferred into new Petri dishes. This procedure was repeated for several times until the microalgal strains were isolated. Each single colony was then inoculated in Erlenmeyer flasks containing liquid sterilized modified BG11 medium, and cultures were maintained under the same conditions described above. According to published descriptions [73,74,75], the isolated microalgal strain was identified by light and transmission electron microscopy examinations as belonging to the genus *Chlorella* (Chlorophyta).

Other different colonies were also transferred into flasks with liquid medium, but results on preliminary growth tests (not shown) showed that the selected *Chlorella* strain was the most promising one.

### 4.3. Experimental Set Up

Growth and nutrient removal tests were carried out using the microalgae belonging to *Chlorella*-like algae previously isolated. To evaluate the growth ability of the microalga in the effluent, preliminary tests were performed cultivating it in both 100% centrate stream and in sterilized modified BG11 medium (treated and control samples, respectively). All cultures were set up in triplicate in 100 mL Erlenmeyer flasks (50 mL culture volume), maintained in static conditions (22–25 °C; 16/8 h light/dark; PAR = 150 μmol_photons_m^−2^ s^−1^) without CO_2_ supplementation [18,19,36,38]; only manual shaking of cultures was carried out once a day. Algal cultures started with a cell density of 1–1.5 × 10^6^ cells mL^−1^. Growth kinetics and PSII maximum quantum yield were evaluated at 0 (inoculum), 4, 7, and 11 days of cultivation. Based on the results of preliminary growth experiments, for further growth and nutrients removal tests, *Chlorella* cells were cultivated in diluted centrate at increasing concentrations: 50%, 60%, 70%, and 80% (wastewater percentage in tap freshwater; composition of nutrients in the tap water: N-NH_4_^+^, 0.02 mg L^−1^, N-NO_3_^−^, 0.09 mg L^−1^, P-PO_4_^3−^, and 0.05 mg L^−1^—for analytical methods, see Paragraph 4.9), keeping unchanged the other cultivation conditions. Starting nutrient content in diluted effluents are reported in Table 3, and N to P molar ratio was about 17 in all samples.

For this part of experimentation, microalgae were cultivated in 500 mL Erlenmeyer flasks (300 mL of culture volume) under the same conditions as those reported above for preliminary growth tests. Growth and morpho-physiological aspects (cell density, dry biomass yield, PSII maximum quantum yield, photosynthetic pigments content, and cell morphology) of microalgae were investigated during 6-day-long experiments. Cell density and PSII maximum quantum yield were evaluated at 0 (inoculum), 2, 4, and 6 days of cultivation, while biomass yield, photosynthetic pigment content, and cell morphology were evaluated only at the beginning and the end of the experiment. Similarly, the N and P content in the effluents was quantified at 0 (inoculum) and 6 days (end) of experiment.

### 4.4. Growth Evaluations

Cell density, expressed as 10^6^ cells mL^−1^, was monitored by cell counting using a Thoma’s counter chamber (HBG, Giessen, Germany). The cell density was reported on a base 2 logarithmic scale. Cell density values were also used to calculate the growth rates in the exponential phase according to the following Equation (1) [76]:

µ (day^−1^) = (log_2_ N_1_ − log_2_ N_0_)/(t_1_ − t_0_) (1)

where µ is the growth rate, N_1_ is the cell density at time t_1_, N_0_ is the cell density at time t_0_ and t_1_ − t_0_ is the time interval (days). 

Algal biomass production was also evaluated by measuring the dry biomass increment. Known aliquots of culture samples were filtered through pre-dried and pre-weighted GF-C glass-fiber filters (Whatman; 1.2 µm pore size). After the filtration process, filters were dried at 60 °C for 48 h and then weighted. The dry biomass yield (expressed as g_DW_ L^−1^; DW, dry weight) was calculated using the following formula (2):

(2) $$Dry\;biomass\;yield\;\left(g/L\right)=\frac{\left(W_{1}-W_{0}\right)}{V}$$

where *W*_1_ is the weight (g) of filters with sample (gross weight), *W*_0_ is the weight (g) of pre-dried filter without sample (tare) and *V* is the filtered sample volume (L).

### 4.5. PSII Maximum Quantum Yield Measurements

In parallel to growth analyses, the PSII maximum quantum yield (*F_V_*/*F_M_*) of algae was measured with a pulse amplitude modulated fluorimeter (Junior PAM, Heinz Waltz GmbH, Effeltrich, Germany). For analyses, aliquots of samples were harvested by centrifugation at 9400× *g* for 10 min. Then, the pellets were placed on pieces of wet filter paper [77] and incubated in darkness for 15 min. After dark-incubation, the samples were exposed to far-red light for 5 s to fully oxidize the electron transport chain [78], and the minimum fluorescence value (*F_0_*) was immediately measured using the measuring light (ML). Subsequently, the maximum fluorescence (*F_M_*) value was measured flashing the sample with saturating light pulse (SP). Considering that the variable fluorescence (*F_V_*) is the difference between *F_0_* and *F_M_* (*F_M_*-*F_0_*) [79], the *F_V_*/*F_M_* ratio was then calculated.

### 4.6. Extraction and Quantification of Photosynthetic Pigments

During algal growth and nutrients removal tests, to determinate the photosynthetic pigments content (chlorophyll *a*, Chl*a*; chlorophyll *b*, Chl*b*; carotenoids, Car), aliquots of samples were harvested by centrifugation at 9400× *g* for 10 min. The pigment extraction was then performed using 100% methanol at 80 °C for 15 min [80]. Then, the extracts were manipulated under dim-green light to avoid photo-degradation, clarified by centrifugation, and measured using a Pharmacia Biotech Ultrospec^®^2000 UV/Vis Spectrophotometer (1-nm bandwidth; Amersham Biosciences, Piscataway, NJ, USA) at 666 nm (Chl*a*), 653 (Chl*b*), 470 nm (Car), and 750 nm (background interferences). Quantification was performed using the equations proposed by Wellburn (1994) [81]. Pigment content was calculated on a cell basis and expressed as nmol_pigment_ 10^−6^ cells.

### 4.7. Light and Fluorescence Microscopy

During experiments, morphological characteristics of microalgal cells were observed with a Zeiss model Axiophot photomicroscope equipped with conventional light and fluorescence attachments. An HBO 100-W pressure mercury vapor lamp (filter set, BP436/10, LP470) was employed as the light source for Chl fluorescence observations. Pictures of algal cells were taken using a Canon Powershot S40 digital camera (4 megapixels), mounted on the ocular lens through a Leica DC 150 system (Leica Camera AG, Solms, Germany). Images were used to determinate the cell sizes with ImageJ freeware.

### 4.8. Transmission Electron Microscopy (TEM) and X-ray Microanalysis

After 6 days of cultivation in diluted centrate streams for nutrients removal tests, microalgal cells were harvested by centrifugation at 600× *g* for 10 min and prepared for transmission electron microscopy according to standard procedures reported [33,72]. Ultrathin sections were observed using the Zeiss EM910 transmission electron microscope available at the Electron Microscopy Centre (University of Ferrara).

To perform energy dispersive X-ray microanalysis (EDS) associated with the scanning electron microscope, samples prepared for TEM were used. Thin sections (1–2 µm) of the samples were mounted on a coverslip, after drying at room temperature, and placed on a metal holder (stub). The prepared samples were then coated with graphite by evaporation with an Emitech K950. Observation was carried out at 20 kV with a Zeiss Evo 40 scanning electron microscope (SEM) with lanthanum hexaboride source and equipped with an Oxford Instruments INCA 300 system for X-ray microanalysis.

### 4.9. Nitrogen and Phosphorus Removal Efficiency

For the evaluation of nutrients removal, NH_4_^+^-N, NO_3_^−^-N, and PO_4_^3−^-P concentrations in the effluents were determined before and after microalgal cultivation on three replicas. Analyses were carried out on the media separated from the algal biomass by centrifugation (5000× *g*, 10 min), and quantification of nutrients was performed using a flow-injection autoanalyzer (Flowsys, Systea SpA, company, Roma, Italy) following standard colorimetric methods [82]. The percentage of nutrients removal efficiency (*RE*) was calculated using the following Equation (3):

(3) $$RE\;\left(\%\right)=\left[\left(C_{i}-C_{f}\right)/\;C_{i}\right]\;\times \;100$$

where *C_i_* and *C_f_* are initial and final nutrients concentrations in culture medium, respectively [83].

### 4.10. Statistical Data Treatments

For each analysis, three biological replicates were set up and means ± standard deviation (s.d.) for *n* sample were calculated. Microsoft Excel was used for construction of graphs. In order to compare control and treated samples in the preliminary growth experiment, a two-tails Student’s *t* test with a significance level of 0.05 was applied using Microsoft Excel tools. Data collected during phytoremediation tests were treated as follows: (i) for growth kinetics and *F_V_*/*F_M_*, at each experimental time, data were directly treated with one-way ANOVA analysis, followed by Tukey’s post hoc test using Origin^®^ 2019 software (significance level, 0.05 for both ANOVA and post hoc tests); homogeneity of data was ascertained using a Brown–Forsythe test (significance level, 0.05) before performing one-way ANOVA; (ii) for dry biomass yield, and for photosynthetic pigments and nutrient contents, data at the inoculation time (time 0) were compared to those after 6 days of cultivation, using a two-tails Student’s *t* test with significance level of 0.05. For Student’s *t* test, the significance levels of differences are shown in the graphs as follows: * *p* < 0.05, ** *p* < 0.01, and *** *p* < 0.001.

## 5. Conclusions

The autochthonous microalgal strain *Chlorella* sp. isolated from effluents of the sludge dewatering process is able to remove nutrients, mainly P, from centrate. However, results from this study also confirm that feasibility of cultivating microalgae in centrate depends on the effluent composition, making it necessary to investigate the optimal concentration of effluent for phytoremediation when using microalgae. Nevertheless, the removal of P-PO_4_^3−^ in particular was very efficient in only 6 days of treatment, especially in cultures where the effluent was used at the highest concentration. This result makes it interesting to test the isolated strain of *Chlorella* in a prototype integrated within the WWTP, not so much with a perspective of obtaining a water that can be directly released into the receiving waterbodies (the thresholds imposed by the European Directives do not allow this; threshold for total P is <1 mg L^−1^), but a water that contains very low levels of P and, thus, when reintroduced into the WWTP, contributes to a more efficient water treatment process: for example, internal recycling of waters with low P would limit the use of chemical agents for P removal in secondary treatments in a sort of more virtuous cycle. In addition, the finding that the algal biomass after WW remediation was enriched in carotenoids and P, compounds of high biotechnological interest which can be valorized with an economic profit, further increases the interest in making the overall management of the depuration system more cost-effective using microalgae-based phytoremediation systems integrated into conventional WWTP.

## Figures and Tables

**Table 1:** Characterization of the centrate streams sampled during summertime (July 2019) and in wintertime (January 2020). Analytical methods employed for each analysis and measurements units are also reported. N to P molar ratio was about 128 and 9.5 in summer and winter samples, respectively.

Parameter	Unit	Analytical Method	Summertime	Wintertime
Total nitrogen	mg N/L	UNI EN 12260: 2004	132.6	338.7
Ammonium	mg NH_4_^+^-N/L	APAT CNR IRSA 4030 A1 Man 29 2003	191.0	508.9
Nitrate	mg NO_3_^−^-N/L	APAT CNR IRSA 4020 Man 29 2003	<0.5	2.3
Nitrite	mg NO_2_^−^-N/L	APAT CNR IRSA 4050 Man 29 2003	<0.04	<0.04
Total phosphorus	mg P/L	UNI EN ISO 15587-2: 2002 ISO 17294-2: 2016+ UNI EN	3.3	119.3
COD	mg O_2_/L	ISO 15705 par 10.2: 2002	606	452
BOD-5	mg O_2_/L	APHA Standard Methods for the Examination of Water and Wastewatered 23rd 2017 5210	110	210
Total suspended solid	mg/L	APAT CNR IRSA 2090 B Man 29 2003	132	114
Al	mg/L	UNI EN ISO 15587-2: 2002 + UNI EN ISO 17294-2: 2016	0.41	0.45
Cr	mg/L	UNI EN ISO 15587-2: 2002+ UNI EN ISO 17294-2: 2016	<0.02	<0.02
Cr (VI)	mg/L	APAT CNR IRSA 3150 C Man 29 2003	<0.02	0.07
Cu	mg/L	UNI EN ISO 1187-2_2002 + UNI EN ISO 17294-2: 2016	<0.005	0.016
Hg	mg/L	UNI EN ISO 1187-2_2002 + UNI EN ISO 17294-2: 2016	<0.0002	<0.001
Pb	mg/L	UNI EN ISO 1187-2_2002 + UNI EN ISO 17294-2: 2016	<0.005	<0.005
Ni	mg/L	UNI EN ISO 1187-2_2002 + UNI EN ISO 17294-2: 2016	0.03	<0.01
Zn	mg/L	UNI EN ISO 15587-2: 2002 + UNI EN ISO 17294-2: 2016	<0.01	0.03

**Table 2:** Ammonium, nitrates, and phosphates concentrations in the filtered centrate (sampling period: January 2020). N to P molar ratio was about 17.

Ammonium	Nitrate	Phosphate
mg NH_4_^+^-N/L	mg NO_3_^−^-N/L	mg PO_4_^3−^-P/L
197.2	0.31	25.9

**Table 3:** Nutrients (N-NH_4_^+^, N-NO_3_^−^, and P-PO_4_^3−^; mg L^−1^) content of winter-season centrate after filtration and dilution with freshwater (50, 60, 70, and 80% centrate in freshwater). In all cases, N to P molar ratio was about 17.

Diluted Effluent	Substrate Composition (%)	Ammonium (mg NH_4_^+^-N/L)	Nitrate (mg NO_3_^−^-N/L)	Phosphates (mg PO_4_^3−^-P/L)
50%	50% effluent + 50% freshwater	98.6	0.63	12.95
60%	60% effluent + 40% freshwater	121	0.61	17.1
70%	70% effluent + 30% freshwater	146.8	0.49	19.77
80%	80% effluent + 20% freshwater	170.6	0.39	22.44

## Data Availability

The datasets generated during and/or analyzed during the current study are available from the corresponding author on reasonable request.

## Supplementary Materials

The following supporting information can be downloaded at: https://www.mdpi.com/article/10.3390/plants12051027/s1, Figure S1: Image at the transmission electron microscope of the isolated alga, selected for phytoremediation tests. The cell shows the characteristic chloroplast containing a large pyrenoid and stromatic starch granules, typical of *Chlorella* sp. algae. P, pyrenoid; * stromatic starch granules. Bar: 1 µm.
