# Supplementary material for: Enhancing Urban Wastewater Treatment through Isolated Chlorella Strain-Based Phytoremediation in Centrate Stream: An Analysis of Algae Morpho-Physiology and Nutrients Removal Efficiency
Source: Plants (Basel). 2023 Feb 24;12(5):1027. doi: 10.3390/plants12051027 (PMC10004828; doi:10.3390/plants12051027)

Figure S1. Image at the transmission electron microscope of the isolated alga, selected for phytoremediation tests. The cell shows the characteristic chloroplast containing a large pyrenoid and stromatic starch granules, typical of *Chlorella* sp. algae. P, pyrenoid; *, stromatic starch granules. Bar: 1 μm.

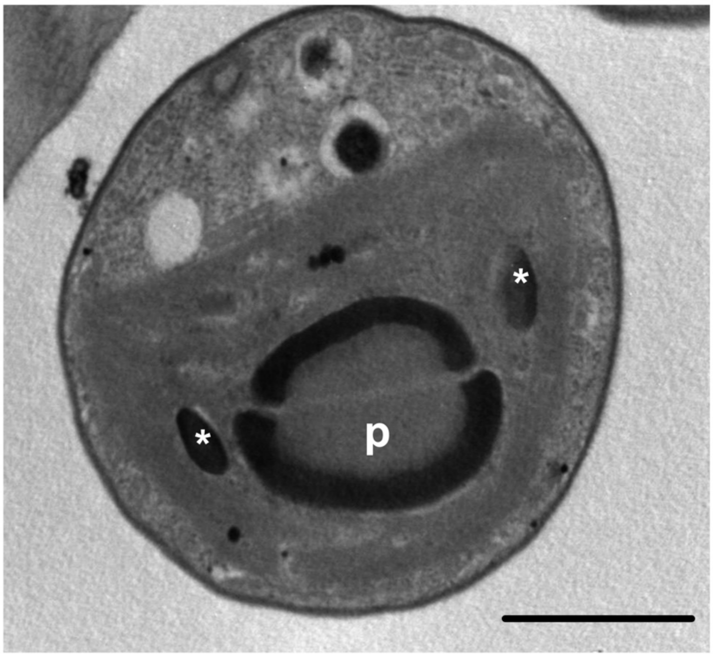

Supplement: Supplementary file 1 [file plants-12-01027-s001.zip › plants-2222887-supplementary.pdf]
